# Sex Does Not Affect the Colour, Shear Stress, and Lipid Oxidation of Pork Meat, but Feed-Added Plant-Derived Extracts, Storage Time and Packaging Type Do

**DOI:** 10.3390/foods12081720

**Published:** 2023-04-20

**Authors:** Begoña Panea, Guillermo Ripoll

**Affiliations:** 1 Animal Science Area, Agrifood Research and Technology Centre of Aragon (CITA), Avda. Montañana 930, 50059 Zaragoza, Spain; 2 Agrifood Institute of Aragon-IA2 (CITA-Zaragoza University), Miguel Servet 177, 50013 Zaragoza, Spain

**Keywords:** feedstuff, packaging type, storage time, lipid oxidation, texture, sex

## Abstract

Essential plant oils added to products, packaging or animal feed are used as a method of preserving food quality because they extend the shelf-life of meat due their antioxidant and/or antimicrobial capacity. This action can be achieved with the correct packaging that preserves the meat’s quality and safety. This study investigates the effects of plant-derived extracts (PDE) on the meat quality and shelf-life of pork packaged in vacuum or modified atmosphere packaging (MAP). Thirty-six barrows and thirty-six gilts were allocated into three experimental groups: the control, garlic extract (1 kg/ton of feed) and oregano–rosemary oil (2 kg/ton of feed) with the same base-diet. Two packaging were used: vacuum and a commercial MAP (70% O_2_, 30% CO_2_). The meat fat content, pH, colour, TBARS values and Warner–Bratzler shear stress were investigated. The sex of the animals did not affect any of the studied variables, whereas PDE affected some of the colour variables and the shear stress; both the packaging type and the storage time affected the colour variables, lipid oxidation and shear stress. Vacuum-packed meat was more stable in terms of colour, lipid oxidation and shear stress than MAP-packed meat.

## 1. Introduction

Meat is an important component of a healthy diet [1], and pork is one of the most consumed meats in the world, with around 122 million tonnes consumed in 2021 [2]. Pork meat’s quality depends on several characteristics such as pH, water holding capacity (WHC), colour or oxidative stability, which determine both technological and sensory quality. Unacceptable quality influences consumer behaviour and can cause high economic losses in the meat industry. On the other hand, the meat industry has been damaged by food panics such as the occurrence of microbial outbreaks, or antibiotic and growth-promoting substance abuses [1]. Therefore, the industry faces the challenge of providing safe food with a shelf-life long enough to guarantee an acceptable sensory quality.

Meat deteriorations are due mainly to lipid and protein oxidation processes and microbial contamination. Oxidation processes cause loss of flavour, colour, and nutritive value. The oxidative stability of meat depends upon the balance of anti- and pro-oxidants present on the muscle; therefore, the use of antioxidants is an effective way to minimise or prevent lipid oxidation, thereby maintaining the nutritional quality and extending shelf-life [3]. In this way, several strategies have been developed to incorporate antioxidants into meat, including the supplementation of animal diets with antioxidant compounds [1]. Synthetic antioxidants have been extensively used in the past, but they exhibit toxic properties [4] and cause rejection by consumers. Therefore, the undesirable effects of synthetic antioxidants have led to greater interest in obtaining natural antioxidant substances.

It has been widely demonstrated that herbs and their essential extracts have antioxidant and/or antimicrobial capacity when they are added to animals’ feedstuff [5,6,7]. Therefore, different plants are used for almost all livestock species [8,9,10,11,12,13]. Nevertheless, diet modifications can causes changes in the degree of saturation of intramuscular fat, and therefore, meat may be prone to lipid oxidation during ageing, which also affects the meat colour [14,15]. Meat colour affects perceptions of freshness, and it is used by consumers to determine a meat’s shelf-life [16,17]. In addition, a direct transfer of aroma components from feed to meat can occur, thereby influencing the sensory qualities [18].

On the other hand, to avoid microbial contamination, meat is often retail packed to preserve its quality and safety [19,20]. Vacuum packaging increases the shelf life of meat by reducing microbial growth [21], but the meat develops a dark brown colour that is rejected by consumers. Modified atmosphere packaging (MAP) maintains the meat’s desirable red colour and prevents bacterial growth, but it also promotes increased lipid oxidation and toughening during storage [22].

In previous experiments carried out in our research centre [23,24], the possibility of reducing the prevalence of *Salmonella* spp. in pigs through diet management was investigated. The current experiment is part of a project regarding the use of plant-derived extract for this purpose [25]. Thus, the aim of this study was to investigate if the effect of the addition of plant-derived extracts to pigs’ feedstuff in extending the meat shelf-life of pork depends upon the animal’s sex and/or packaging type.

## 2. Materials and Methods

### 2.1. Animals and Handling

The current paper is part of a series concerning the same experimental design. Previously published papers are available [26,27].

The used procedures were approved by the Institutional Animal Care and Use Committee of the Research Centre (Procedure number 2011-03). A total of 72 Duroc x (Landrace x Large White) animals were randomly allocated into three feeding groups: control, garlic, and oil. The feed for all animals was a cereal mixture composed of corn, soya, wheat, barley, and rapeseed which was given ad libitum. Pigs were housed in 80% slotted floor pens (3.50 m × 3.00 m) in a natural environment barn and had free access to a pelleted diet and water throughout the trial. The diet was formulated to ensure the requirements of pigs were met [28], and consisted of corn, sunflower, soya, rapeseed, wheat and barley. Dry matter was from 89.2–89.5% depending on the rearing step, whereas crude protein was from 14% to 16%, crude fibre was 4.6–5.5% and non-digestible fibres was 16.8–19.7%. A detailed composition can be seen in the work of Panea and Ripoll [27].

Animals from the control group were fed only this diet, whereas the other two groups were fed with the diet added with each plant-derived extracts (from now, PDE) from initiation to slaughter. In the garlic group, 1 kg of a garlic (Garlic sativum) complex (Garlicon ^®^ Domca, S.A.U., Granada, Spain), was added to each ton of feed, resulting in a 25 g/ton combination of propyl propane thiosulfonate/propyl propane thiosulfonate added to their feed. In the oil group, each ton of feed was supplemented with 2 kg of a compound (Repaxol ^®^, Molimen, Barcelona, Spain) formed by a mixture of carvacrol (from oregano), thymol (from thyme), cinnamic aldehyde (from cinnamon) and eugenol (from clove), which was microencapsulated by a lipidic matrix and added to the feed.

Animals were slaughtered at the weight described by Protected Geographic Indication “Jamón de Teruel” (hot carcass weight > 86 kg), at an EU-authorized slaughterhouse (Calamocha, Spain). The animals were electrically stunned (225 to 380 V/0.5 A for 5 to 6 s), exsanguinated, scalded, skinned, eviscerated, and split down the midline following commercial practices. The carcasses were kept at 4 °C for 24 h, and then 24 carcasses from each experimental group (12 barrows and 12 gilts) were randomly selected; subsequently, left loins from the 5th thoracic vertebra to the 6th lumbar vertebra were excised and transported to our laboratory. At 24 h *post mortem*, the pH of all loins was measured at the 5th thoracic vertebra level with a pH-metre equipped with a Crison 507 penetrating electrode (Crison Instruments S.A., Barcelona, Spain), with temperature compensation. Thereafter, the muscles *Longissimus thoracis et lumborum* were deboned and sliced to obtain the samples described below.

### 2.2. Sample Preparation and Packaging

A completely randomized design was used. One-centimetre-thick steak was obtained for intramuscular fat measurement, and freeze-dried extraction was carried out in a VirTis Wizard 2.0 lyophilizer (SP Scientific, Gardiner, NY, USA) for 7 days at −50 °C and 13.332 Pa, and kept at −20 °C until analysis. The meat was weighed before and after freeze-drying to calculate the dry matter.

For assessing meat colour, five 3.5 cm steaks were taken. The first was used to measure the initial point, that is, the basal colour profile without the packaging effect but considering the sex effect. The second and third steaks were vacuum-packed and kept in the dark at 4 °C for 4 days or 7 days, respectively. The chosen vacuum bags (PA/PE 20/70, Coimbra Pack, S.L., Coimbra, Spain) had an oxygen permeability ≤ 80 cm^3^·m^−2^·24 h^−1^ at 1 atm and a water vapor permeability ≤ 2 g·m^−2^·24 h^−1^. The third and fourth steaks were packed in an MAP tray with a 2:1 gas:meat ratio and headspace and a commercial blended atmosphere (70% O_2_, 30% CO_2_, Praxair España, Madrid, Spain). The cover film (Cryovac 1825–50, Cryovac Europe, Barcelona, Spain) had an oxygen permeability of 14.8 cm^3^·m^−2^·24 h^−1^ at 1 atm and a water vapor permeability of 16 g·m^−2^·24 h^−1^. Then, the samples were kept in the dark at 4 °C for 4 days or 7 days, respectively.

After colour measurement, all the samples were vacuum-packed as described above, frozen at −20 °C, and used to determine the lipid oxidation level.

To determine the shear stress, another five different 3.5 cm steaks were obtained. As with the colour measurement, the first one was used to determine the initial shear stress without the packaging effect but considering the sex effect. For vacuum packaging, two 3.5 cm steaks were vacuum-packed as described above, kept in the dark at 4 °C for 4 days or 7 days, respectively, and frozen at −20 °C until analysis. For MAP, two other 3.5 cm steaks were packed as described above, kept in the dark at 4 °C for 4 days or 7 days, respectively, and frozen at −20 °C until analysis.

**Figure 1:**
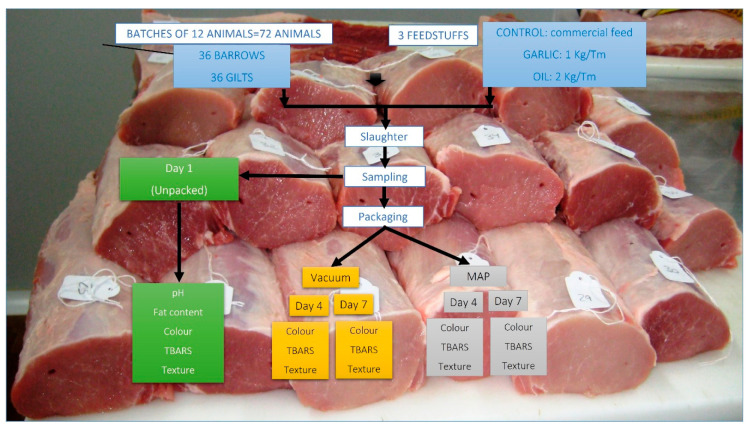
Experimental design. A completely randomized design was used.

A resume of the experimental sampling and design are given in Figure 1.

### 2.3. Instrumental Procedures

The fat content was quantified using the Ankom Procedure (AOCS Am 5-04) with an Ankom extractor (Model XT10, Ankom Technology, Madrid, Spain).

All the samples used for colour were allowed to bloom for 30 min. The colour was measured with a Minolta CM-2006d spectrophotometer (Konica Minolta Holdings, Inc, Osaka, Japan) in the CIELAB space (CIE, 1986) with a measured area diameter of 8 mm; the specular component included 0% UV; a standard illuminant D65, which simulates daylight (colour temperature 6504 K); a 10° observer angle; and zero and white calibration. The lightness (L*), redness (a*) and yellowness (b*) were recorded, and the hue angle (H_ab_) and chroma (C_ab_) indices were calculated as H_ab_ = tan^–1^(b*/a*), expressed in degrees, and C_ab_ = (a*^2^ + b*^2^)^1/2^ [29].

The lipid oxidation was determined following Bertolín, et al. [30]. This method is based on extraction with trichloroacetic acid (TCA), reaction with 2-thiobarbituric acid (TBA) and quantification with ultraperformance liquid chromatography with a fluorescence detector (UPLC-FLD) with λ_xcitation_ = 530 nm and λ_emission_ = 550 nm and with a diode array detector (UPLC-DAD) with λ_absorbance_ = 532 nm. TBARS values are expressed as milligrams of MDA per kilogram of muscle.

For the shear stress analysis, samples were thawed overnight at 4 °C in the dark, and then they were heated in a 75 °C water-bath to an internal temperature of 70 °C; this was monitored with a Testo thermocouple equipped with a probe (Testo SE & Co. KGaA, Lenzkirch, Deutschland). A minimum of 10 subsamples of a 10 × 10 mm^2^ cross-sections were obtained following longitudinal configuration [31]. Samples were sheared using an Instron 5543 (ITW Test & Measurements, Buckinghamshire, UK) fitted with a Warner–Bratzler device. The shear stress (N/cm^2^) was recorded [32].

### 2.4. Statistics

Statistical analyses were performed using XLStat 17.03 software. A completely randomized design was used. Two general linear model (GLM) procedures were carried out; the first investigated the effects of the sex and PDE addition on the pH and fat amount. The second one determined the influence of sex, PDE, storage time (4 or 7 days) and packaging type (vacuum or MAP) on the colour, lipid oxidation and texture. Means and standard errors were calculated with Tukeys’ test for significant differences (*p* < 0.05).

## 3. Results and Discussion

### 3.1. pH and Intramuscular Fat Content

The *p* values for the effect of the sex and PDE on the pH and intramuscular fat content are in Table 1. Neither PDE nor sex affected the pH or the intramuscular fat percentage values, and no interactions were found between effects.

All the pH values were in a normal range for pig meat and agreed with several authors who described values from 5.6 to 5.8 at 24 h post-mortem [33,34,35,36]. The lack of an effect of dietary supplementation with PDE on the pH agreed with the findings of other authors. For example, Ranucci, et al. [37] used a commercial mix composed of equal parts of oregano essential oil and sweet chestnut wood extract, and found no effect on pH either at 45 min post-mortem or at 24 h post-mortem. Similarly, O’Grady, et al. [1] used grape seed and bearberry and concluded that pH was unaffected by supplementation. On the other hand, the absence of sex effect on pH was also reported by several authors in animals of similar characteristics [34,35]. Therefore, the pH will affect neither colour nor texture.

The current intramuscular fat percentage values (4.0% on average) agreed with those of several authors for similar animals. Garitano, Liebana, Feliz, Daza and López [36] reported 3.4% in Duroc x (Landrace x Large White) crosses, whereas Morales, et al. [38] reported the values of 3.2% for Pietrain, 4.2% for Duroc and 4% for Large White breeds, and Calvo Díez, et al. [39] reported a value of approximately 3.6% for females slaughtered at 130 kg. The absence of the sex effect on intramuscular fat percentage was reported previously by Latorre, et al. [40] in animals with 2.5–2.8% of intramuscular fat and by Simitzis, Symeon, Charismiadou, Bizelis and Deligeorgis [11] in animals with 1.2–1.4% of intramuscular fat. Nevertheless, Correa, Faucitano, Laforest, Rivest, Marcoux and Gariépy [34], working with Duroc x (Landrace x Large White) animals with 1.6–2.2% of intramuscular fat, stated that intramuscular fat content was higher in barrows than in gilts, although the differences were more noticeable at 107 kg liveweight or at 125 kg liveweight than at 115 kg liveweight at slaughter. Since our animals weighed around 112 kg, this could partially explain the lack of sex effect in current experiment. In addition, the diets of the current experiment were iso-energetic and iso-proteic, and all the animals had the same carcass weight (around 112 kg).

The absence of an influence of the PDE on the intramuscular fat amount agrees with the conclusions of other authors in experiments with different levels of oregano oil [11] or a mix of oregano and sweet chestnut [37].

### 3.2. Meat Colour

All colour variables were affected by packaging type (*p* < 0.001) and, except for the H_ab_, by the storage time (*p* < 0.001). In addition, significant interactions (*p* < 0.05) were found between packaging type and storage time for all colour variables except H_ab_. PDE affected only a*, b* and C_ab_, and no interactions with the other factors were found. Finally, sex had no effect on colour variables, and no significant interaction with the other factors were found (*p* > 0.05).

**Figure 2:**
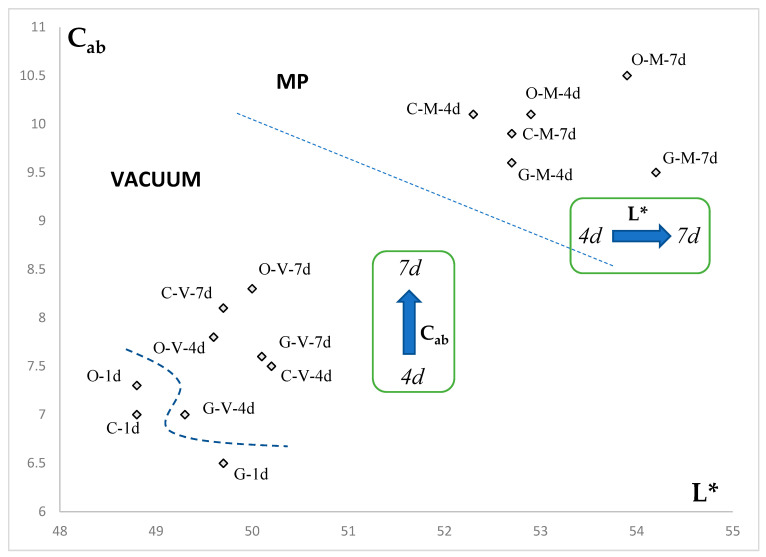
Representation of C_ab_ vs. L* variables in function of the PDE, packaging type and storage time. C.—control batches; G.—garlic batches; O.—oil batches: V.—vacuum samples; M.—MAP samples; d.—day.

Since PDE affected some of the variables, we calculated the means for the interaction of packaging and time for the PDE groups, and these data are in Appendix A. To facilitate the understanding of the results, we depicted a map with C_ab_ vs. L* values (Figure 2). We chose these two variables because several studies [16,41,42] have shown that when consumers make their buying decision based on colour, these two variables are the ones that best predict their behaviour, so algorithms can be established to explain their choice, and it is possible to create rules that include L* and C_ab_ as discriminating variables. In the bottom left corner, we can find these values at 1 day. Next to them are all the vacuum values; the MAP values are in the bottom right corner of the picture. The first conclusion we can draw is that the colour of vacuum-packed meat is less luminous and less intense than the colour of MAP-packed meat, as expected. On the other hand, in vacuum packaging, C_ab_ values increased from the 4th to the 7th day, that is, with the storage time, the meat colour became more intense. However, in MAP packaging, L* values increased when storage time did, with or without changes in the C_ab_ values. Finally, we observe that for each of the packaging types and storage times, the values from the garlic batch are lower than the values from the other PDE groups.

The current values are in accordance with those that were reported by several authors regarding animals of similar characteristics [34,35,40,43]. The lack of a sex effect on colour variables has been reported by several authors [11,34,35,39,40,43,44].

Regarding diet effect, some authors have reported no effect of oregano or garlic [18], rosemary [45] or oregano oil [11] dietary supplementation on the meat colour. Nevertheless, other studies [37] have stated an absence of the effect on raw meat but an effect on cooked meat, showing that plant- extract batches exhibit the lowest L* values and the highest a* values. The present results show that PDE influenced meat colour, but since it affects only a* and b*, and their influence is much less than that of the packaging type or storage time, it can be considered that the changes caused by the PDE addition should not cause the problem of rejection by the consumer; from a practical point of view, they should not affect purchase intentions. In fact, in a previous study of the series [27], we investigated the effect of PDE, time exposure and packaging type on consumer visual appraisal and found that PDE did not affect to consumers’ purchase intention and that the 66,8% of the consumers would buy the meat, especially during the two first days of exposure. In addition, meat packed in MAP had in general higher visual scores than meat packed in vacuum, which could be linked with the higher values for L* found in current experiment.

The effect of packaging types has been extensively described in the literature. High-oxygen atmospheres are widely used to maintain a bright red colour, which is attractive for consumers; however, oxygen concentrations above 50% can promote lipid oxidation, resulting in meat discolouration [46]. The packaging type particularly affected redness (a*), which is higher in MAP packaging than in vacuum packaging. Therefore, the resulting dark and less intense colour of meat is one of the main drawbacks of vacuum packaging [47,48,49].

Concerning storage time, the L* increased from the 1st day until the 7th day, but this increase was more noticeable in MAP than in vacuum packaging. The present results agreed with the conclusions presented by other authors. So, in a study on vacuum-packed meat from castrated males, Álvarez-Rodríguez, et al. [50] showed that L* increased from the 5th day of ageing, and C* increased from the 3rd day of ageing. Similarly, Alonso, et al. [51], who employed meat that was frozen, unfrozen and MAP-packed thereafter, reported that L* increased from time 0 to the 9th day of exposure.

### 3.3. Lipid Oxidation

Lipid oxidation was affected only by packaging (*p* < 0.001) and storage time (*p* < 0.001), and an interaction was found between them (*p* = 0.014), whereas neither sex (*p* = 0.352) nor PDE (*p* = 0.959) affected the oxidation level, and no other interactions between effects were found (*p* > 0.05). The values of MDA as a function of packaging and storage time are given in Table 2.

The value at 1 day was, as expected, lower than the others. Nevertheless, whereas there was no change from the 4th to the 7th day in vacuum packaging, the values increased throughout the storage time in MAP packaging. At both the 4th and 7th days, MAP presented the highest oxidation values.

Current values agree with the data reported by several authors. Ramírez and Cava [52], working with 3 different Iberian x Duroc genotypes, reported values of MDA from 0.063 to 0.073, whereas Hallenstvedt, et al. [53], working on Norwegian Landrace_Yorkshire) x Duroc crosses fed with diets containing fish oil, showed values from 0.06 to 0.08. In a study with Landrace x Large white x Duroc animals fed with a standard cereal mixture and with meat packed in MAP, Alonso, Muela, Gutiérrez, Calanche, Roncalés and Beltrán [51] showed values of approximately 0.072 at day 0 and approximately 0.5 at day 9 of exposure, which was a large increase in MDA over time.

Several authors [11,51,52,53], working with animals of different breeds and reared under different dietary regimes, reported no sex effect on the TBARS values, which is in accordance with the current results. In addition, in the current experiment, we did not find a sex effect on intramuscular fat content, which partially explains the lack of the sex effect on lipid oxidation.

The effect of the amount and increase in oxidation products in pork during storage is extensively described in the literature because it is one of the main mechanisms of quality decline in meat products, which is one of the most important problems concerning acceptability of meat to consumers; therefore, it is very important for the industry. Álvarez-Rodríguez, Villagrasa, Ros-Freixedes, Gol, Henríquez, Pena, Estany and Tor [50] reported that in vacuum-packed meat from Duroc animals fed with cereals, the MDA values did not change throughout the first 7 days of storage. Similarly, Spanos, et al. [54] reported that after 7 days of storage, samples packed in MAP with 50% oxygen showed significantly higher values of MDA than samples packed with 0% O_2_. Nevertheless, Ramírez and Cava [52], working with meat packed in a polystyrene tray wrapped with PVC plastic, reported no changes over the time in lipid oxidation, which implies that in non-protective packaging, the maximum level of oxidation is reached from the beginning, thereby reinforcing the importance of packaging fresh meat.

Concerning the diet effect, in the literature, the effect of adding plant extracts or components has shown contradictory results. Simitzis, Symeon, Charismiadou, Bizelis and Deligeorgis [11] reported no effect of dietary administration of oregano essential oil on the oxidation level, and they concluded that the components of oregano were probably not introduced into the cell membranes. In the same way, Janz, Morel, Wilkinson and Purchas [18] reported no effect of the oil source (rosemary, garlic, oregano or ginger) on the lipid oxidation level. Other authors have found an effect of rosemary extract plus ascorbic acid on the lipid stability of sausages [55] and an effect of a herbal mixture (of *Salvia officinalis*, *Urtica dioica*, *Melissa officinalis* and *Echinacea purpurea*) on the lipid stability of pork after 5 months of freezing [56]. Finally, Fernandez-Lopez, et al. [57] reported that garlic’s antioxidant properties depend on the concentrations and the sample extraction method, which could explain the differences between studies and the lack of PDE addition in lipid oxidation in the current study.

It has been reported that the threshold value for sensory detection is approximately 0.50 mg MDA/g tissue in pork [4], which is much higher than that of the current results. This, together with the lack of effect of the PDE, implies that for practical purposes, the addition of PDE should not pose any threat to the commercialization of meat.

### 3.4. Shear Stress

The shear stress was affected by the PDE (*p* < 0.0001) and packaging type (*p* = 0.023), and significant interactions were found between packaging type and PDE (*p* = 0.044), and between storage time and PDE (*p* = 0.004), whereas sex had no effect on the shear stress (*p* > 0.005) and no additional significant interactions between effects were found.

**Figure 3:**
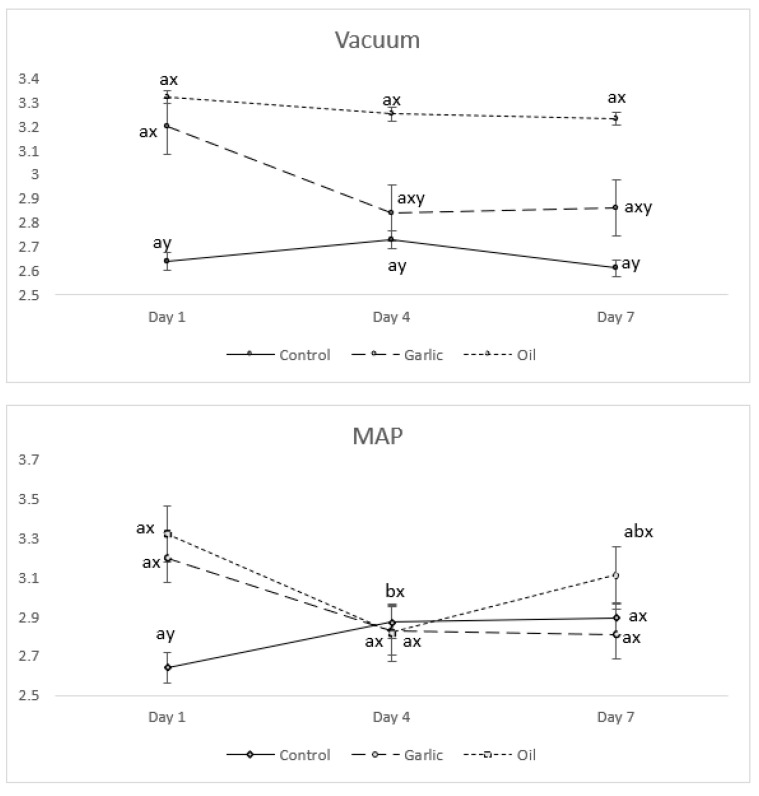
Shear stress values as a function of the PDE, packaging type and storage time, a, b.—differences between days (*p* < 0.05), for a certain PDE; x, y.—differences between PDE (*p* < 0.05) for a certain day. Bars represents the standard error.

The interaction between PDE and storage time is because in vacuum packaging, time did not affect the shear stress values, whereas in the MAP packaging, it did. So, we represented the results of the function of the packaging separately in order to clearly see the PDE and storage time effects. The results are shown in Figure 3.

In the vacuum-packed group, apart from the absence of an effect of the storage time, as expected, differences between PDE were found (*p* < 0.005). Independently of the storage time, the oil batch presented the highest values, followed by garlic batch, with the control batch presenting the lowest values. Nevertheless, the behaviour in MAP is not so clear. Neither on the 4th day nor the 7th day of storage time was a difference in PDE found, whereas on day 1, the control batch presented the lowest values. On the other hand, neither the control batch nor the garlic batch were found to be affected by storage time, whereas in the oil batch, values decreased from the day 1 to the day 4, then increased again thereafter. So, from a practical point of view, vacuum packaging guarantees a constant hardness, whereas MAP is unpredictable, and additional studies would be necessary.

The current values for the shear stress are in accordance with those reported in the literature for animals of similar characteristics. Calvo Díez, Rodríguez Sánchez and Latorre Górriz [39] reported a shear stress value of 2.89 kg in females slaughtered at 130 kg. Latorre, Ripoll, García-Belenguer and Ariño [35] found values from 2.5 to 3 kg in animals belonging to P.G.I. Teruel, and Álvarez-Rodríguez, Villagrasa, Ros-Freixedes, Gol, Henríquez, Pena, Estany and Tor [50] reported values of 2.5 kg in barrows.

The lack of a sex effect on shear stress has been reported previously [11,35,44,51], although some authors [40] reported that females had tougher meat than barrows.

Sørheim, Wahlgren, Nielsen and Lea [47] reported that meat was more tender if it was vacuum-packed instead of packed in the presence of oxygen, especially during the first 7 days, because in a vacuum, there is a continuous enzymatic action; however, in MAP, the protein oxidation process results in an increase in carbonyl compounds which form di-sulphur links, resulting in tougher meat [58]. In addition, Bell, et al. [59] concluded that O_2_, and not CO_2_, was the gas responsible for inadequate tenderization in packed meat, which could explain the results found in the current experiment for MAP.

Various authors have reported no effect on meat’s textural characteristics when adding several PDEs. Janz, Morel, Wilkinson and Purchas [18], working with diets enriched with rosemary, garlic, oregano, or ginger extracts, reported no effect of the supplementation, although the values for rosemary and garlic were around 20% lower than values for the control batch (around 80 N and 100 N, respectively). Similarly, Simitzis, Symeon, Charismiadou, Bizelis and Deligeorgis [11], in a study with oregano addition, and Ranucci, Beghelli, Trabalza-Marinucci, Branciari, Forte, Olivieri, Pazmay, Cavallucci and Acuti [37], comparing oregano and sweet chestnut wood extracts, reported no differences between groups; they attribute this to the absence of any differences in protein and intramuscular lipid contents. Nevertheless, in the current experiment, the oil batch tended to present higher values than the other groups, indicating that both the type and the level of supplementation influence the meat traits [11], and indicating the necessity of further studies.

## 4. Conclusions

Under the conditions of the present experiment, it can be concluded that the sex of the animals did not affect any of the studied variables, whereas PDE affected some of the colour variables and the shear stress; both the packaging type and storage time affected the colour variables, lipid oxidation and shear stress.

Vacuum-packed meat had a less intense colour and lower L* values than MAP-packed meat. With the storage time, the meat became yellower in vacuum packaging and lighter in MAP packaging. The garlic batch presented the lowest values for L* and C_ab_.

Lipid oxidation did not change over the time in vacuum packaging, but increased throughout the storage time in MAP packaging, and MAP values are always higher than vacuum values.

The shear stress values did not change over time in vacuum packaging, but did in MAP packaging, showing an initial decrease and a subsequent increase.

From practical purposes, the addition of PDE should not cause any problem for the commercialization of meat but, further studies are desirable in order to understand meat’s interactions with its packaging type.

## Figures and Tables

**Table 1:** The *p* values and global means for the effect of the sex and plant-derived extract addition on the pH and intramuscular fat content.

	Global Mean	s.e.	PDE Effect (D)	Sex Effect (S)	DxS Effect
pH	5.60	0.008	0.161	0.115	0.963
Intramuscular fat percentage (IMF)	4.04	0.175	0.372	0.181	0.650

**Table 2:** The means and standard error (s.e.) for lipid oxidation (mg MDA/g) as a function of packaging type and storage time.

	Day 1 (Starting Point)	Day 4	Day 7	s.e.
Vacuum	0.066 c	0.072 c	0.072 c	0.0017
MAP		0.097 b	0.111 a	0.0022
s.e.	0.0029	0.0020	0.0027	

a, b, c.—different letters imply differences between groups (packaging type*storage time).

## Data Availability

Data sharing is not applicable to this article.

## Appendix A. The Means, Standard Error (s.e) and *p* Values for Colour Variables in Function of the PDE, Packaging Type and Storage Time

**Control Batch**									
		Vacuum		MAP					
	1 day	4 days	7 days	4 days	7 days	s.e.	Packaging (P)	Time (T)	PxT
L*	48.8 b	50.2 ab	49.7 b	52.3 a	52.7 a	0.29	0.010	0.008	0.098
a*	3.2 b	3.5 b	3.8 b	5.3 a	5.1 a	0.15	<0.0001	0.006	0.262
b*	6.2 b	6.6 b	7.1 b	8.5 a	8.4 a	0.13	<0.0001	<0.0001	0.037
C_ab_	7.0 b	7.5 b	8.1 b	10.1 a	9.9 a	0.69	<0.0001	<0.0001	0.039
H_ab_	63.3	62.3	62.8	58.6	59.0	0.17	0.042	0.400	0.911
Garlic Batch									
		Vacuum		MAP					
	1 day	4 days	7 days	4 days	7 days	s.e.	Packaging (P)	Time (T)	PxT
L*	49.7 b	49.3 b	50.1 b	52.7 ab	54.2 a	0.31	<0.0001	0.007	0.275
a*	2.9 b	3.1 ab	3.3 ab	4.9 a	4.5 ab	0.19	0.028	0.115	0.130
b*	5.6 c	6.1 bc	6.7 b	8.1 a	8.3 a	0.15	<0.0001	<0.0001	0.139
C_ab_	6.5 b	7.0 b	7.6 b	9.6 a	9.5 a	0.19	<0.0001	<0.0001	0.059
H_ab_	64.6	65.2	65.8	60.1	62.6	1.21	0.780	0.793	0.320
Oil Batch									
		Vacuum		MAP					
	1 day	4 days	7 days	4 days	7 days	s.e.	Packaging (P)	Time (T)	PxT
L*	48.8 b	49.6 b	50.0 b	52.9 a	53.9 a	0.34	0.001	0.001	0.002
a*	3.3 b	3.7 b	3.9 b	5.5 a	5.4 a	0.15	<0.0001	0.001	0.144
b*	6.5 b	6.8 b	7.2 b	8.5 a	8.9 a	0.15	<0.001	<0.001	0.009
C_ab_	7.3 b	7.8 b	8.3 b	10.1 a	10.5 a	0.18	<0.0001	<0.0001	0.006
H_ab_	53.4	61.6	61.3	57.1	59.1	0.70	0.019	0.160	0.724
a, b, c.—differences between storage times for a certain PDE batch.									
